# Trial History Effects in Stroop Task Performance Are Independent of Top-Down Control

**DOI:** 10.1371/journal.pone.0039802

**Published:** 2012-06-27

**Authors:** Monicque M. Lorist, Jacob Jolij

**Affiliations:** 1 Department of Experimental Psychology, University of Groningen, Groningen, The Netherlands; 2 BCN-NeuroImaging Center, University Medical Center Groningen, University of Groningen, Groningen, The Netherlands; Baycrest Hospital, Canada

## Abstract

In this study we sought to elucidate what mechanisms underlie the effects of trial history on information processing. We explicitly focused on the contribution of conflict control and S-R binding to sequential trial effects. Performance and brain activity were measured during two hours of continuous Stroop task performance. Mental fatigue, known to influence top-down processing, was used to elucidate separate effects via top-down and bottom-up mechanisms. Here we confirm that performance in the Stroop task is indeed strongly modulated by stimulus history. Performance was affected by the kind of advance information available; dependent on this information adjustments were made, resulting in differential effects of cognitive conflict, and S-R binding on subsequent performance. The influence of mental fatigue on information processing was mainly related to general effects on attention.

## Introduction

Fortunately, we are generally able to effectively ignore irrelevant information and to selectively attend to task relevant information, which is crucial for adequate performance. However, every now and then our cognitive system might function less efficiently, allowing irrelevant information to interfere with information processing. Such transitory deteriorations of our attention system and related performance decline have, for example, been associated with mental fatigue [1], [2], [3]. In selective attention tasks, for example, participants have to identify targets at relevant locations, but ignore targets at irrelevant locations. Well-rested participants actively orient attention towards relevant stimulus features, as measured with event-related brain potentials (ERPs); however, fatigue causes this active orienting of attention to deteriorate [1]. In other words, with increasing mental fatigue relevant information seemed to be extracted less efficiently and irrelevant information interfered more strongly with adequate task performance.

One of the cognitive tasks that have been used frequently to examine the role of interference is the Stroop task [4], [5]. In this task individuals have to name the colour in which a word is presented, while ignoring the meaning of the word. Stroop task performance was found to be dependent on the congruency between colour and word meaning. If they do not match (e.g., the word green printed in red) responses are slower and the number of errors is higher compared to performance in trials in which the colour of a word is congruent with the meaning of that word.

Performance differences between congruent and incongruent stimuli are generally attributed to interference between the outcomes of two different processing routes. In these so-called “dual-route” models [6], [7] it is assumed that relevant information is processed via a controlled, top-down route, activating a response according to task instructions specifying stimulus-response translation rules. Via a second, bottom-up route information might automatically activate an alternative response. Strength of processing in the automatic route is dependent on natural or learned stimulus-response relations. In case of an incongruent Stroop stimulus, irrelevant word meaning which is processed via the automatic route is associated with a response different to the response associated with the relevant colour feature, eliciting cognitive conflict. Controlled processing involved in resolving this conflict takes time, which might explain the slower responses on incongruent trials compared to congruent trials. Based on previous findings indicating that selective attention mechanisms are functioning less efficiently in mentally fatigued individuals [1], one might hypothesise that distracting information in Stroop stimuli, processed via the second, automatic route might interfere more strongly in fatigued individuals, affecting behaviour negatively.

Performance in a Stroop task not only depends on congruency of the current stimulus, it was found to be modulated by congruency of the preceding stimulus, as well. More specifically, the Stroop interference effect was found to be reduced if stimuli occurred immediately after an incongruent trial, compared with occurrence after a congruent trial [8]. Different models have been put forward to explain these trial sequence effects. According to the influential conflict monitoring model [9] sequence effects result from cognitive conflict elicited in previous trials. Cognitive conflict elicited in incongruent Stroop trials is detected by the anterior cingulate cortex (ACC), which in turn signals a need for greater cognitive control to the prefrontal cortex (PFC). The PFC is involved in the actual implementation of appropriate adjustments in control in order to resolve conflicts and increase performance efficiency on subsequent trials [8], [10]. Enhanced levels of control during trials following incongruent stimuli result in a stronger attentional focus on task-relevant stimulus features, thereby reducing the interference of irrelevant stimulus information in these trials. As a result conflict in incongruent trials will be reduced and responses will speed-up. On the other hand, facilitation by congruent irrelevant features (i.e. congruent word meaning) will diminish, resulting in slower responses in congruent trials preceded by incongruent trials. Congruent trials are not associated with conflict and therefore do not lead to adjustments in cognitive control and related modifications of subsequent behaviour. Thus, according to the conflict monitoring model, sequence effects result from conflict-based top-down adjustments in cognitive control.

In addition to the stronger interference of irrelevant information with increasing mental fatigue due to less efficient attentional control, as mentioned above, Lorist and colleagues [11], [12] found that in mentally fatigued subjects the detection of conflicts was hampered and performance adjustments after error trials were inadequate. They manipulated the level of conflict in a modified Simon task by assigning right hand responses either to stimuli presented at the right side of fixation (low conflict) or at the left side of fixation (high conflict [12]). A left hand response was assigned vice versa. An ERP component reflecting level of response conflict, occurring if a stimulus activates more than one response, is the fronto-central N2 [13], [14], [15], [16], [17], [18]. Boksem et al. [12] found that the amplitude of the N2 was decreased in the high conflict condition in fatigued subjects and differences between high and low conflict conditions disappeared with increasing mental fatigue, indicating that fatigued participants were less aware of cognitive conflicts.

In Stroop task performance the N2 component might manifest as a negative deflection with a later onset than the N2 observed in other cognitive control studies [15], [19], [20]. This Stroop-N2 or N450 component has a similar fronto-central scalp distribution and is modulated by cognitive control in a similar way as the N2. In addition to the N450, a sustained parietal slow positive potential (SP) was found to be modulated by conflict processing [19], [20]. The SP starts approximately 500 ms post-stimulus and is more pronounced following high conflict trials than low conflict trials. Although both the N450 and the SP were found to be sensitive to cognitive conflict, Larson et al. [21] reported that particularly the SP was sensitive to the previous trial context. If indeed sequential trail effects are driven by conflict detection, one might expect that with time on task adjustments in performance will disappear and N450/SP amplitude will not be modulated by trail sequence in fatigued participants.

Although the conflict monitoring model has been quite influential, the role of conflict in performance adjustments across trials has been questioned [22]–[24]. An alternative model put forward to explain sequential trial effects is the feature integration model [22]. This model posits that sequence effects are related to the formation of stimulus–response (S-R) associations; if specific stimulus and response features co-occur in time these features are integrated into a common episodic memory representation. Subsequent activation of any one of these features automatically activates the related feature, which creates a substantial performance benefit in S–R repetition trials. However, partial repetitions, in which one feature changes but the other remains the same, are processed slowly, because recently formed association between stimulus and response features become inappropriate in the new context, resulting in cognitive conflict which increases reaction times (RTs). On the other hand, faster responses are expected in complete alternation of stimulus and response features, because in this condition no previous feature coupling has to be overcome. Thus, the feature integration theory suggests that the influence of previous stimuli on current behaviour is mediated by bottom-up associative priming mechanisms rather than top-down adjustments due to conflict related changes in cognitive control. Mental fatigue has been found to primarily influence top-down control, while automatic processes seem relatively insensitive to mental fatigue [2], [11], [25], [26]. Therefore, if sequence effects during the Stroop task indeed rely on the relative automatic formation of S-R associations, effects of mental fatigue on performance and related brain activity are expected to be minimal. Although the conflict monitoring model emphasises the role of conflict in sequential performance adjustments and the feature integration theories stresses associative priming, these top-down and bottom-up mechanisms do not seem to be mutually exclusive [27], [28]. Both mechanisms may act in concert via different routes in our information processing system. However, the relative contribution of top-down and bottom-up influences remain elusive, partly because a great number of studies examining sequential trial effects have primarily focussed on the role of conflict in sequence effects. In these studies the influence of confounding factors was controlled for by analysing only a subset of trials (e.g., no-repeat trials in the Kerns et al. study [8]) or differences between specific feature repetition conditions were ignored [28], making it more difficult or even impossible to examine the influence of these alternative mechanisms on trial sequence effects. In the present study we explicitly sought to evaluate the relative contribution of both top-down control processes and bottom-up associative priming mechanisms to sequential trial effects. Therefore, a three colour version of the Stroop task was used that allowed the examination of the relation between feature repetitions and congruency sequences, avoiding confounds between both factors [23], [27]. Instead of analysing a sub-set of trials, all stimulus categories were taken into account and specific feature repetition conditions was added as a factor in the analysis. Moreover, the inclusion of a third response alternative allowed us to evaluate S-R binding more precisely. According to the feature integration theory, a stimulus repetition primes a response repetition by biasing the competition towards the previous response bound with the repeated stimulus [29]. In case two response alternatives are available stimulus alternation implies inhibition of the previous response, resulting in a bias towards the alternative response. The three-response-version allowed us to actually examine whether previous responses are indeed inhibited in alternation trials. It is hypothesised that the threshold to trigger a response will be lower for responses which are not inhibited compared to responses that are inhibited, which will result in incorrect responses mainly consisting of those response alternatives which are not inhibited. According to the conflict monitoring model a different pattern of responses is expected in error trials. Conflict elicited in the previous trial modulates the attentional bias in the current trial. Therefore more errors will be expected due to interference of irrelevant information in those trials preceded by low conflict trials compared to trials preceded by high conflict trials.

In summary, in this study we examined behavioural and electrophysiological indices of cognitive control to elucidate the contribution of both top-down conflict control and bottom-up S-R binding on sequential trial effects during Stroop task performance. Mental fatigue, induced by time on task, was used in order to manipulate top-down control. The influence of mental fatigue on Stroop task performance is expected to be most evident if sequential performance adjustments are based on top-down processes.

## Methods

### Ethics Statement

The study was approved by the Medical Ethical Committee of the University Medical Center of Groningen and the experiment was undertaken in compliance with national legislation and the Declaration of Helsinki. Participants gave written informed consent before participation.

### Participants

Eighteen undergraduate students (5 women; M_age_ = 20.7 years (SD = 2.1)) from the University of Groningen (Groningen, The Netherlands) participated in this study. All of them were right-handed, had normal or corrected-to-normal visual acuity and adequate colour vision. They did not work night shifts, reported to have normal sleep patterns, and did not use prescription medication.

### Stimuli

A Stroop task was used in which participants had to react to the colour used to print colour names. Stimuli were presented in the centre of the visual field on a colour monitor positioned at 80 cm from the subject’s eyes. On each trial, a word indicating a colour name (“rood” (red), “blauw” (blue) or “groen” (green); font: Courier New, size: 24, style: bold) printed in red, blue or green was presented for 200 ms on a black background. During inter-stimulus intervals a fixation mark (a white plus sign) was visible in the centre of the visual field. Inter-stimulus intervals varied randomly between 1500 and 2000 ms (steps of 100 ms). Responses were provided by pushing a predefined button on a response box. Three different versions of button-colour combinations were used (Latin square design), so that each colour-button combination occurred in the experiment. Button-colour versions were assigned randomly to participants.

Half of the stimuli were congruent (e.g., the word red printed in red), the other half was incongruent (e.g., the word red printed in blue). The combination of congruency of the presented stimulus and congruency of the previous stimulus led to the existence of four sequence conditions, a congruent stimulus followed by another congruent one (CC), a congruent stimulus followed by an incongruent stimulus (CI), an incongruent stimulus followed by a congruent stimulus (IC), and an incongruent stimulus followed by another incongruent one (II). The stimuli were semi-randomly presented, so that each sequence of stimuli occurred about equally often.

### Procedure

The experimental session started around 1.00 p.m. and lasted three hours. After the participant arrived at the laboratory electrodes were applied and the procedure was explained, without giving specific information about the duration of the experimental task. Thereafter, the subject was seated in a dimly illuminated, sound-attenuated, and electrically shielded room. Before the experimental task started, a short practice block of 5 minutes was performed in order to check whether participants had no problems perceiving stimuli and understood the instructions correctly. The experimental task during which behaviour and the electroencephalogram (EEG) was measured, took about 120 minutes in total. Participants were instructed to respond as quickly and accurately as possible to the colour of a word (while ignoring the meaning of the word) with a button press to one of three colour-coded response keys using the index, middle, and ring fingers of their right hand. After task performance electrodes were removed and subjects were debriefed.

### EEG Recordings

The EEG was recorded, using Sn electrodes attached to an electrode cap (ElectroCap International), positioned according to the standard 10–10 system [30]. The electrodes were referenced to electronically linked earlobes. The electro-oculogram (EOG) was recorded bipolarily with Sn electrodes from the outer canthi of both eyes and above and below the left eye. The Ag/AgCl electrode for earthing the subject was placed on the sternum. Electrode impedances were reduced to less than 5 kΩ. Signals were recorded using a TMSI Refa common reference amplifier with a 20 bits resolution. The signals were amplified with a time constant of 10 s, low pass filtered at 30 Hz and digitized at a rate of 250 Hz.

### Data Reduction and Statistical Analyses

Mean RTs were calculated for trials in which a correct response was provided between 150 and 1500 ms (93.3% (SD = 3.1) of the trials). Mean error rate was quantified as the percentage of incorrect responses between 150 and 1500 ms within each condition. Trials in which no response was given were regarded as misses (1.2% (SD = 1.5) of the trials).

ERP analysis was performed using the Vision Analyzer (Brain Products GmbH) software package. For ERP analyses trials containing amplifier saturation artefacts were excluded from analysis. Ocular correction was performed using the Gratton and Coles method [31]. Average ERPs were computed separately for each electrode position for the correct trials in the different conditions. The averaging epoch started 100 ms prior to stimulus onset and lasted until 900 ms post-stimulus. The averages were aligned to a 100 ms pre-stimulus baseline. For further analysis mean amplitude for frontal (F7, F3, Fz, F4, F8), central (T7, C3, Cz, C4, T8), and parietal (P7, P3, Pz, P4, P8) electrodes was calculated in 17 periods of 50 ms each, from 50 to 700 ms post-stimulus.

Behavioural data and mean amplitudes of the ERPs in the different intervals were entered as dependent variables to SPSS univariate analysis of variance for repeated measures. Analyses which showed violation of the assumption of sphericity were adjusted using the ε∼*-adjustment procedure recommended by Quintana and Maxwell [32]. For clarity, uncorrected degrees of freedom values are presented in the results section. If the main analysis indicated a significant interaction (α≤.05) between factors, follow-up analyses were performed using Bonferroni adjustments. Within-subject factors for the behavioural analysis were time on task (4 intervals of 30 min), congruency (congruent/incongruent) and congruency of the previous stimulus (sequence: congruent/incongruent). To examine the influence of feature repetitions within each of the four sequence/congruency conditions (i.e. CC, CI, IC and II; see Table 1), additional analyses were performed with repetition condition added as within-subject factor (levels were dependent on the specific congruency/sequence condition). For ERP analysis, anterior-posterior electrode position (antpos: frontal, central, parietal) and laterality of electrode position (lat: from left to right (e.g., F7, F3, Fz, F4, F8)) were added as factors.

**Table 1:** Stimulus types.

Congruency(current trial)	Sequence condition	Feature(s) repeated	Repetition condition	Percentage trials within category
Congruent	Congruent-Congruent	Colour and word	CC_C+W+_	12.5
Congruent	Congruent-Congruent	No-repeat	CC_C−W−_	12.5
Incongruent	Congruent-Incongruent	Colour	CI_C+W−_	8.3
Incongruent	Congruent-Incongruent	Word	CI_C−W+_	8.3
Incongruent	Congruent-Incongruent	No-repeat	CI_C−W−_	8.3
Congruent	Incongruent-Congruent	Colour	IC_C+W−_	8.3
Congruent	Incongruent-Congruent	Word	IC_C−W+_	8.3
Congruent	Incongruent-Congruent	No-repeat	IC_C−W−_	8.3
Incongruent	Incongruent-Incongruent	Colour and word	II_C+W+_	6.25
Incongruent	Incongruent-Incongruent	Colour	II_C+W−_	6.25
Incongruent	Incongruent-Incongruent	Word	II_C−W+_	6.25
Incongruent	Incongruent-Incongruent	No-repeat	II_C−W−_ *	6.25

* This condition contains trials in which the colour of the previous stimulus is word meaning of the present stimulus (II_C−W−_^a^), trials in which word meaning of the previous stimulus forms the colour of the present stimulus (II_C−W−_^b^), and trials in which the colour reappears as word and word meaning reappears as colour of the current stimulus (II_C−W−_^c^).

## Results

First, the observed behavioural and EEG effects of the congruency and time on task manipulations are described based on trial level analyses. Thereafter, we focus on sequential trial effects. We first report results of the analysis in which congruency of the previous trial is taken into account. Thereafter we concentrate on the effects of feature repetitions (dependent on sequence condition colour and word-repeat, colour-repeat, word-repeat and no-repeat can be discerned) within the congruency sequence conditions (CC, CI, IC and II).

### Trial Level Analysis

**Figure 1:**
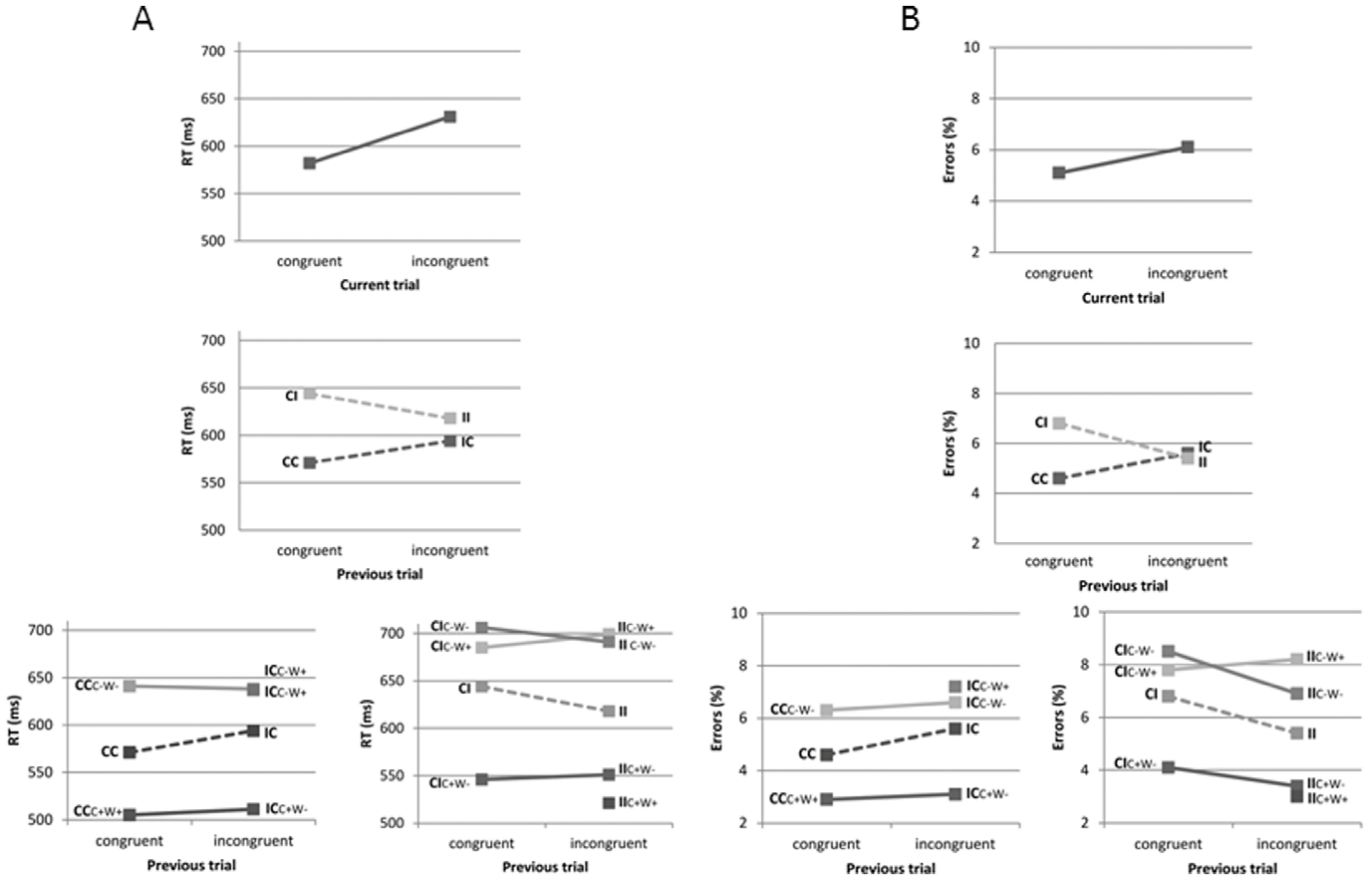
RTs (a) and error rates (b). RTs (ms; a) and error rates (% incorrect responses; b) for the different stimulus categories (see Table 1 for explanation of abbreviations). Top panel depicts trial level congruency effects, the middle panel depicts sequential trial effects for the different congruency conditions and in the lower panel results of the different feature repetition conditions are shown. Percentage errors are expressed relative to the number of trials within the specific stimulus category.

As expected, faster responses (582 ms, SD = 48) and lower error rates (5.1%, SD = 2.6) were observed in congruent Stroop trials compared to incongruent trials (RT: 631 ms (SD = 54); error rate: 6.1% (SD = 3.2); congruency: F(1,17) = 157.67, p<.001 and F(1,17) = 10.36, p = .005, for RT and error rate, respectively; upper part of Figure 1a and 1b), reflecting the Stroop interference effect. Distraction due to word meaning was observed in the pattern of button presses in error trials; participants more often pressed the incorrect response button corresponding to word meaning of the stimulus (58% of the errors) than the response button associated with none of the stimulus features present in the current trail (42% of the errors; F(1,17) = 22.56, p<.000).

**Figure 2:**
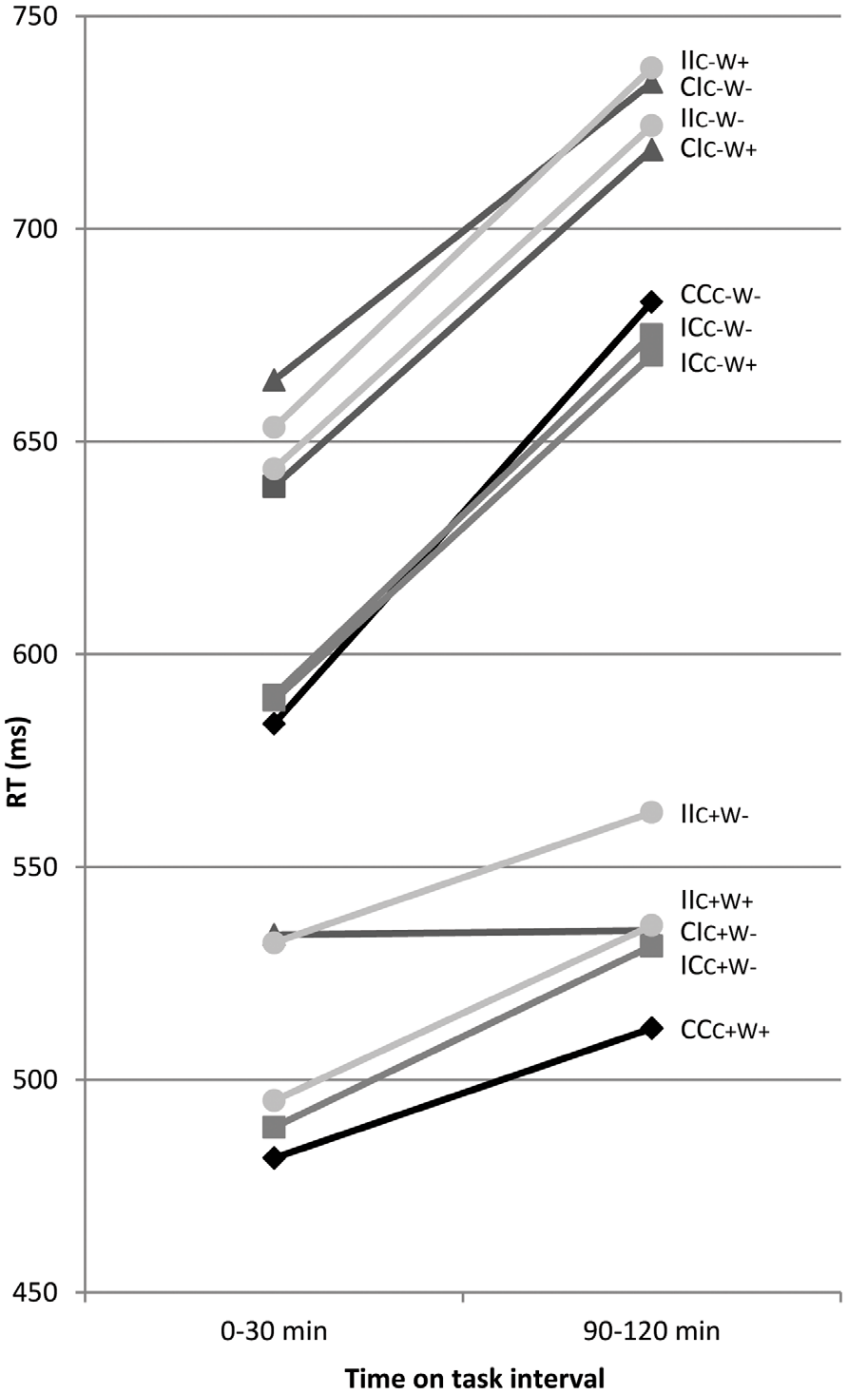
ERPs for different congruency conditions. Average ERPs recorded from Fz, Cz and Pz, superimposed for the congruent (solid lines) and incongruent condition (dotted lines). ERPs are averaged over time on task conditions.

ERPs elicited after the presentation of congruent and incongruent Stroop stimuli showed a consistent pattern of N1, P2, N2, and P3 components (Figure 2). Differences between congruent and incongruent trials became significant around 150–200 ms post-stimulus, reflecting a more positive going P2 component in congruent compared to incongruent stimuli (congruency: F(1,17) = 10.14, p = .005).

RTs and error rate increased with time on task (RT: 571 (SD = 63), 598 (SD = 62), 629 (SD = 61) and 629 ms (SD = 53) for the first to the fourth 30 min time on task interval, respectively: F(3,51) = 10.45, p<.001; error rate: 4.7% (SD = 2.5), 5.4% (SD = 2.9), 5.9% (SD = 3.4) and 6.5% (SD = 3.1) for the first to the fourth 30 min interval, respectively: F(3,51) = 6.96, p = .001). This increase was similar for congruent and incongruent trials (time on task x congruency: F(3,51) = 1.45, n.s. and F(3,51) = 1.20, n.s., for RT and error rate, respectively).

**Figure 3:**
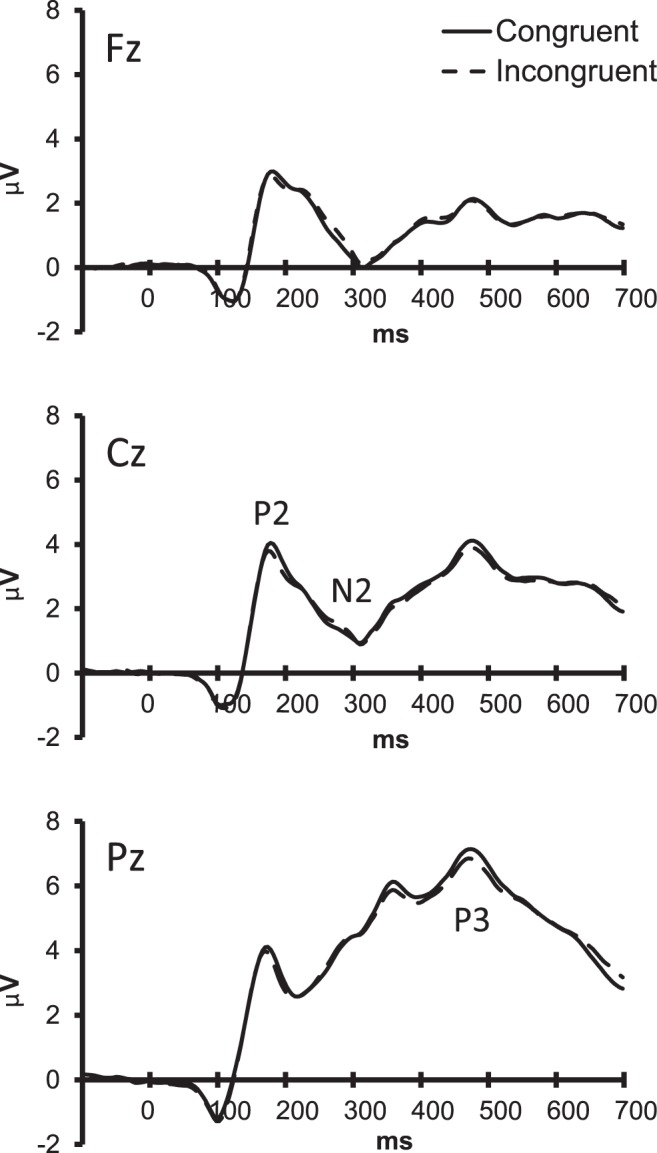
ERPs for different time on task intervals. Average ERPs recorded from Fz, Cz and Pz, superimposed for the four time on task intervals. ERPs are averaged over sequence conditions.

Time on task effects in the ERP became significant around 100–150 ms after stimulus presentation in the form of a decrease in P2 amplitude, which was followed by a clear decrease in P3 amplitude (Figure 3). These changes were most pronounced from the first to the second half hour of task performance, especially at centro-parietal midline sites (time on task between 100–200 and 250–600 ms post-stimulus: F(3,51) = 3.33–42.43, all p’s≤.035; time on task x antpos between 100–200, 300–350, 400–500 and 600–650 ms: F(6,102) = 2.99–6.28, all p’s≤.037; time on task x lat between 100–200 and 250–550 ms: F(12,204) = 3.61–21.68, all p’s≤.018; time on task x antpos x lat between 100–200 and 250–350 ms: F(24,408) = 3.69–8.04, all p’s≤.006).

Consistent with the behavioural results, the ERP effects of time on task were not modulated by the congruency of the current trial, indicating that mental fatigue did affect task performance but not at the level of conflict control.

### Sequential Trial Analysis

#### Conflict sequence conditions

The next analysis took into account the congruency identity of the previous trial. Participants were instructed to respond to the present trial, however, the results indicated that performance was affected by congruency of the previous trial (sequence x congruency: F(1,17) = 71.09, p<.001 and F(1,17) = 47.86, p<.001, for RTs and error rate, respectively; middle part of Figure 1a and 1b); participants reacted faster and more accurate to congruent trials if the previous trial was congruent (571 ms (SD = 47); 4.6% (SD = 2.4)) than if it was incongruent (594 ms (SD = 51); 5.6% (SD = 2.7)). For incongruent trials this pattern was reversed; responses to incongruent stimuli were faster and more accurate after incongruent stimuli (618 ms (SD = 54); 5.4% (SD = 3.1)) than after congruent stimuli (644 ms (SD = 55); 6.8% (SD = 3.4)). The significant sequence by congruency interaction reflected a larger interference effect for trials following a congruent trail than for trials following an incongruent trial, which is a pattern similar to that observed in previous studies [33]. Although these results show that performance is influenced by congruency of the previous stimulus, we found no evidence of interference of irrelevant information on the pattern of errors. As mentioned above, in error trials participants pressed the incorrect response button corresponding to word meaning more frequent than the response button unrelated to one of the stimulus features, however, the level of conflict elicited in the previous trial did not modulate this effect (sequence x response choice: F(1,17) = .63. n.s.).

In addition to the behavioural results, effects were observed in the ERP data; a more negative SP amplitude was elicited in incongruent trials than in congruent trials, which was mainly due to a negative shift in incongruent trials preceded by a congruent stimulus (sequence x congruency x lat: F(4,68) = 5.61–9.81, all p’s≤.005; between 400–500: sequence x congruency x antpos x lat: F(8,136) = 2.86–2.95, all p’s≤.048). The effects of time on task on performance and brain activity were not modulated by congruency of the previous trial, indicating that mental fatigue did not affect sequential trial effects.

#### Feature repetitions

The following sequential trial analyses examined more closely the influence of feature repetitions on the effects described in the previous section. Behaviour and related brain activity within each conflict sequence condition were analysed, with feature repetition condition as factor (i.e. colour and word-repetition, colour-repetition, word- repetition and no- repetition). Note that these analyses were performed for each sequence condition separately, because the specific feature repetition conditions differed across the conditions (see Table 1). In addition, the influence of time on task on these effects is examined.

### Congruent Trials

#### CC condition

As mentioned earlier, RTs to congruent trials preceded by a congruent trial were on average 571 ms. However, it should be realised that two types of stimuli made up this CC category; stimuli which were repetitions of the previous stimulus (both colour and word meaning of the previous stimulus were repeated; CC_C+W+_; see Table 1) and stimuli which did not have features in common with the previous stimulus (CC_C−W−_). Additional analysis showed that participants reacted faster to CC stimuli if the stimuli was a repetition of the previous stimulus (505 ms, SD = 51) than in case no stimulus features were repeated (641 ms, SD = 58); a difference of 136 ms (F(1,17) = 112.20, p<. 001; lower part of Figure 1a). Moreover, error rate was higher in the no-repeat condition (6.3% (SD = 3.3)) than in the repeat condition (2.9% (SD = 1.8); F(1,17) = 36.04, p<.001; lower part of Figure 1b).

**Figure 4:**
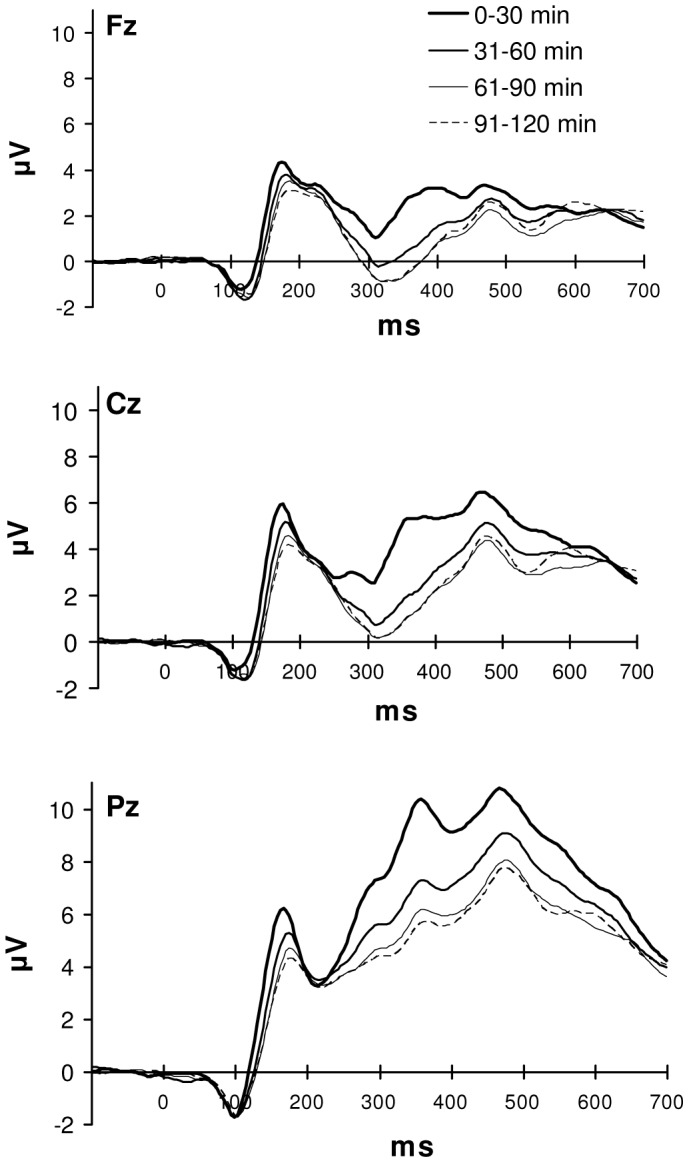
ERPs for different repetition conditions. Average ERPs recorded from Cz and Pz, for the different repetition conditions in each sequence condition (CC: left upper panel; CI: left lower panel; IC: right upper panel; II: right lower panel).

In addition to these behavioural effects, feature repetition condition also affected brain activity (Figure 4; upper left); CC_C+W+_ trials elicited a smaller N450 than CC_C−W−_ trials, especially at Cz. The SP showed a reverse effect and had a maximum at Pz (repetition condition between 350–400 and 600–700 ms: F(1,17) = 5.36–7.86, all p’s≤.033; repetition condition x antpos between 450–700 ms: F(2,34) = 4.65–9.35, all p’s≤.036; repetition condition x lat between 350–450 and 600–700 ms: F(4,68) = 3.89–5.29, all p’s≤.047; repetition condition x antpos x lat between 450–700: F(8,136) = 2.41–4.31, all p’s≤.42).

**Figure 5:**
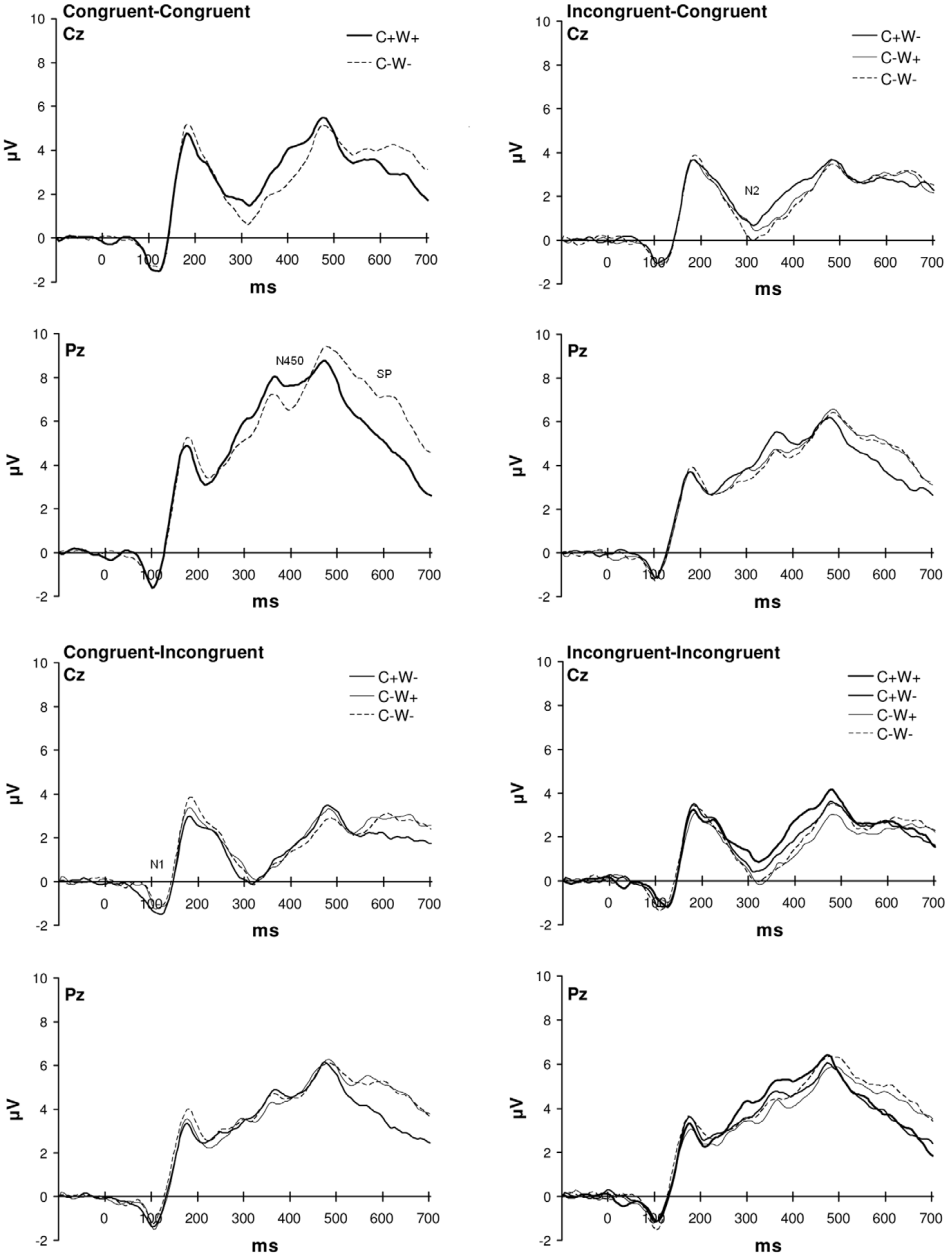
Time on task effects on RT. RTs (ms) superimposed for the different sequence conditions in the first (0–30 min) and fourth time on task interval (90–120 ms; see Table 1 for explanation of abbreviations).

Effects of time on task in the CC trials were dependent on repetition condition, as well. The observed increase in RT with time on task was more pronounced in the no-repeat trials (99 ms from the 1^st^ to the 4^th^ 30-min interval) than in the repetition trials (31 ms from the 1^st^ to the 4^th^ 30-min interval; time on task x repetition condition: F(3,51) = 20.50, p<.001; time on task for CC_C+W+_ trials: F(3,51) = 2.75, n.s.; time on task for CC_C−W−_ trials: F(3,51) = 5.43, p = .003; Figure 5). The fatigue related increase in error rate did not differ significantly between both repetition conditions (3.6% vs. 1.3%, for CC_C−W−_ and CC_C+W+_ trials, respectively; time on task: F(3,51) = 7.29, p<.001; time on task x repetition condition: F(3,51) = 1.96, n.s.). Differences in brain activity between the repetition conditions were not modulated by time on task.

#### IC condition

The IC sequence condition consisted of stimuli in which either colour- (IC_C+W−_), word- (IC_C−W+_) or no stimulus features (IC_C−W−_) of the previous stimulus were repeated. Participants reacted faster and more accurate to the colour-repeat than to word- and no-repeat stimuli (F(2,34) = 110.82, p<.001 and F(2,34) = 35.34, p<.001, for RT and error rate, respectively; see lower part of Figure 1a and 1b). RTs and error rates in the word- and no-repeat condition did not differ significantly.

The repetition-related changes in performance were accompanied by changes in brain activity in the IC trials; modulations of N2 and SP amplitude were observed (Figure 4; upper right). N2 amplitude was less negative in the colour-repeat condition than in the other conditions, which was most pronounced at midline electrode sites (repetition condition between 300–400 ms: F(2,34) = 5.98–10.83, all p’s≤.006; repetition condition x lat between 250–300 and 350–400 ms: F(8,136) = 2.76–5.38, all p’s≤.036). SP amplitude was more negative in the colour-repeat condition compared to the other conditions, which was most pronounced at parietal sites (repetition condition x antpos between 500–700 ms: F(4,68) = 8.91–11.56, all p’s≤.007; repetition condition x lat between 600–700 ms: F(8,136) = 4.64–5.39, all p’s≤.003).

In the word- and no-repeat IC condition a more pronounced increase in RT was observed from the first to the fourth 30-min interval than in the colour-repeat trials (81, 85 and 43 ms for word-repeat, no-repeat and colour-repeat trials, respectively; time on task x repetition condition: F(6,102) = 3.44, p = .004; Figure 5). Prolonged task performance had no differential effect on error rate across the IC repetition conditions (F(6,102) = 1.05, n.s). The observed ERP differences between repetition conditions were not influenced by time on task.

In summary, the repetition of specific stimulus features affected performance and related brain activity in congruent trials. These effects seemed to be independent of sequence condition; in both CC and IC trials colour repetition was associated faster responses and less errors. Moreover, N2 amplitude was decreased, especially at Cz, and the sustained parietal slow positive potential was smaller in the colour-repeat trials than in the other repetition conditions, which seems to provide support for the feature integration model. These conclusions were indeed confirmed in additional analyses directly comparing CC and IC conditions in which colour was repeated (i.e. CC_C+W+_ vs. IC_C+W−_: F(1,17) = 2.45, n.s. and F(1,17) = .58, n.s., for RT and errors, respectively) and no features were repeated (i.e. CC_C−W−_ vs. IC_C−W−_: F(1,17) = 1.57, n.s. and F(1,17) = 1.31, n.s., for RT and errors, respectively). In addition, for both the CC and IC sequence conditions time on task effects were mainly limited to increased RTs in the word- and no-repeat trials. The observed ERP differences between repetition conditions were not influenced by time on task.

### Incongruent Trials

#### IC condition

As for the IC trials, the CI sequence condition consisted of stimuli in which either colour- (CI_C+W−_), word- (CI_C−W+_) or no stimulus features (CI_C−W−_) of the previous stimulus were repeated. Faster and more accurate responses were observed to the colour-repeat than to word- and no-repeat stimuli (see lower part of Figure 1a and 1b). RTs were slowest in the no-repeat trials, while the error rates were similar in the word-repeat and no-repeat conditions (F(2,34) = 109.19, p<.001 and F(2,34) = 18.93, p<.001, for RT and error rate, respectively).

ERPs elicited in CI trials were affected by feature repetition condition, as well (Figure 4; lower part). These effects started somewhat earlier than in the CC and IC trials. ERPs observed at fronto-central sites in CI trials showed an increased N1 amplitude (repetition condition x antpos: F(4,68) = 5.91, p = .005) and a decreased P2 amplitude after colour repetitions compared to the other repetition conditions (repetition condition: F(2,34) = 4.35, p = .021; repetition condition x lat: F(8,136) = 4.35, p = .003). Late effects of repetition condition were observed between 550 and 700 ms, reflecting a more negative going SP in colour-repeat trials, which was maximal at parietal sites (repetition condition: F(2,34) = 5.03–8.59, all p’s≤.021; repetition condition x antpos: F(4,68) = 5.0–8.11, all p’s≤.016; repetition condition x lat: F(8,136) = 2.94–5.03, all p’s≤.038; repetition condition x antpos x lat: F(16,272) = 2.38–3.68, all p’s≤.029).

In the word- and no-repeat CI trials a more pronounced increase in RT was observed from the first to the fourth 30-min interval than in the colour-repeat trials (79, 70 and 33, ms for word-repeat, no-repeat and colour-repeat trials, respectively; time on task x repetition condition: F(6,102) = 7.34, p<.001; Figure 5). The increase in RT with time on task in the CI_C+W−_ condition did not reach the level significance (F(3,51) = 2.15, n.s.). The effect of prolonged task performance on error rate was similar across the different CI repetition conditions (F(6,102) = 1.06, n.s.).

Although in all conditions a clear decrease in ERP amplitude was observed from the first to the second half hour of task performance, in the no-repeat condition the decrease from the second to third interval was more pronounced than in the other repetition conditions, while in the word-repeat condition a more pronounced decrease in ERP amplitude was observed from the third to fourth time on task interval than in the other repetition conditions (time on task x repetition condition between 300–650 ms: F(6,102) = 2.76–3.79, all p’s≤.039).

#### II condition

In the II sequence condition, colour- (II_C+W−_), word- (II_C−W+_), colour *and* word- (II_C+W+_), and neither colour nor word- (II_C−W−_) repetition conditions can be discerned. Formally, the no-repeat II condition contains three subcategories: trials in which the colour of a word forms word meaning of the next stimulus (IIC-W-a), trials in which word meaning forms the colour of the next stimuli (IIC-W-b), and stimuli in which the colour reappears as word and word meaning reappears as colour of the next stimulus (IIC-W-c). Behaviour analysis showed no differences between the these IIC-W- sub-categories (F(2,34) = 2.64, n.s. and F(2,34) = .37, n.s. for RT and percentage errors, respectively), therefore they were pooled into the IIC-W- condition.

RT data showed significant differences between these categories (F(3,51) = 115.35, p<.001). Responses to trials in which both colour and word of the preceding stimulus were repeated were fastest (521 ms). In case the colour of the previous stimulus was repeated RTs were on average 551 ms. The other conditions elicited slower responses which did not differ significantly from each other (699 and 691 ms for the II_C−W+_ and II_C−W−_ condition, respectively; see lower part of Figure 1a and 1b). In addition, participants were more accurate in the II_C+W+_ (3.0%) and II_C+W−_ (3.4%) conditions than in the other repetition conditions (8.2% and 6.9% for II_C−W+_ and II_C−W−_ trials, respectively; time on task x repetition condition: F(3,51) = 15.61, p<.001).

In the II sequence condition ERP showed a less negative N2 amplitude in the colour repeat conditions than in the conditions in which colour was not repeated (repetition condition between 300–400 ms: F(3,51) = 4.99–6.41, all p’s≤.004; repetition condition x lat between 350–450: F(12,204) = 2.92–3.28, all p’s≤.040). The amplitude of the parietal SP was smaller in the colour-repeat trials than in the other conditions (repetition condition x antpos between 500–700 ms: F(6,102) = 5.04–9.30, all p’s≤.010; repetition condition x antpos x lat between 550–700 ms: F(24,408) = 2.76–3.27, all p’s≤.010)).

Effects of time on task on RT differed across the II-sequence conditions (F(9,153) = 3.00, p = .005; Figure 5); a significant increase in RT with increasing fatigue was observed for the word- and no-repeat trials (time on task for II_C−W+_ trials: F(3,51) = 9.51, p<.001 and for II_C−W−_ trials: F(3,51) = 10.83, p<.001), while this effect was non-significant in the II_C+W+_ and II_C+W−_ trials (time on task for II_C+W+_ trials: F(3,51) = 3.13, n.s. and II_C+W−_ trials: F(3,51) = 2.13, n.s.). The fatigue-related increase in error rate was similar across the four II conditions (time on task: F(3,51) = 5.27, p = .003; repetition condition x time on task: F(9,153) = 1.29, n.s.).

Time on task effects on brain activity were mainly related to a decrease in SP amplitude from the first to the second time on task interval with increasing mental fatigue; the timing of which was dependent on repetition condition. An additional negative shift from the third to the fourth interval was observed in II_C+W+_ trials and in trials in which no features were repeated the effects of time on task were primarily reflected in a negative shift from the second to the third 30-min period of task performance (400–500 ms: time on task x repetition condition; F(9,153) = 2.30–2.50, p’s≤.019).

In sum, faster and more accurate performance was observed if the relevant stimulus feature (i.e. colour) was repeated, while the effects of repetition of the irrelevant stimulus feature (i.e. word) were less pronounced compared to the no-repeat conditions. Early ERP effects were observed in incongruent trials preceded by congruent trials at fronto-central sites, indicating that colour repetitions increased the N1 amplitude and decreased the P2 amplitude in this condition compared to the other repetition conditions. The effects of feature repetition on N2 amplitude were mainly found in the II sequence condition. Similar to congruent trials, parietal SP amplitude was less positive in the colour repeat trials than in the other repetition conditions in the incongruent trials. With time on task SP amplitude decreased; the timing of this effect differed across the different repetition conditions.

**Figure 6:**
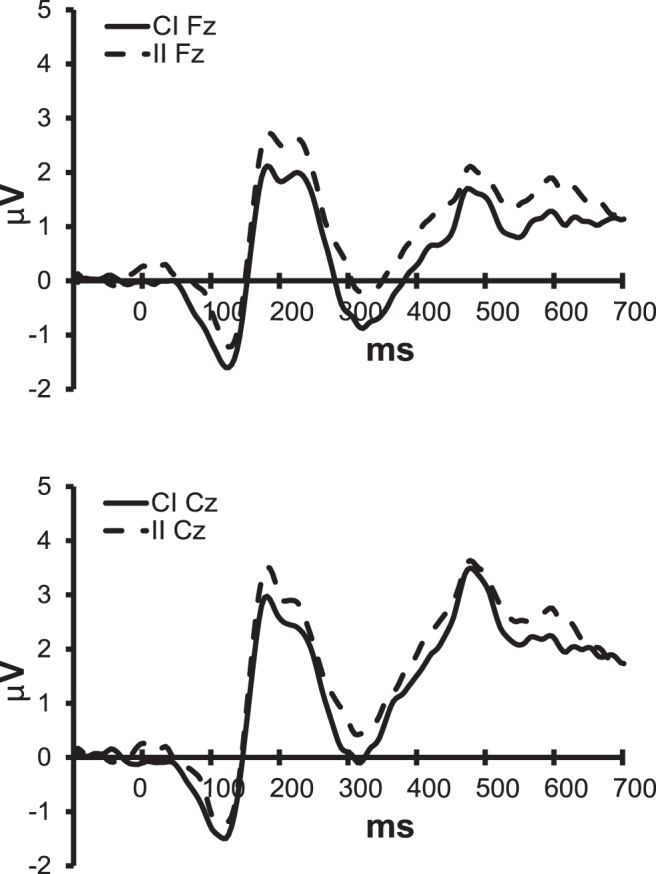
ERPs for the CI (solid lines) and II (dotted lines) colour-repeat sequence conditions. Average ERPs recorded at Fz and Cz.

The observed differences between CI and II trials suggest that congruency of the previous trial modulated performance on incongruent Stroop trials. Comparison of CI and II conditions in which colour was repeated showed that RT and error rate in these trials were not modulated by the congruency condition of the previous trial (CI_C+W−_ vs. II_C+W−_: F(1,17) = 1.98, n.s. and F(1,17) = 3.55, n.s. for RT and errors, respectively). However, ERP data did show differences between both conditions (Figure 6). Fractionated modulations were observed at fronto-central sites; colour-repeat incongruent stimuli preceded by colour-repeat congruent trials elicited a more pronounced negative going ERP than stimuli preceded by incongruent trials, which reached the level of significance between 150–350, 500–550 and 600–650 ms post-stimulus (stimulus type (CI_C+W−_, II_C+W−_) x lat between 150–200 ms: F(4,68) = 3.506, p = .040; stimulus type x antpos between 200–350, 500–550 and 600–650 ms: F(2,34) = 4.08–5.32, all p’s≤.032).

In the word-repeat condition congruency of the previous trial had an effect on performance; RTs in word-repeat trials were faster if the previous trial was congruent (685 ms) compared to an incongruent preceding trial (699 ms; CI_C−W+_ vs. II_C−W+_: F(1,17) = 10.85, p = .004). Note that this sequential trial effect is opposite to what is expected based on the conflict adaptation model. These behavioural effects were not accompanied by changes in brain activity. If no features were repeated congruency history had neither an effect on performance nor on ERPs.

Dutzi and Hommel [29] argued that the response bound to the previous stimulus is inhibited in subsequent stimulus alteration trials. Our results confirmed this suggestion; erroneous responses which were repetitions of responses required on the previous trial occurred less frequent than the alternative response option (27% vs. 73%, (F1,17) = 44.77, p<.000), supporting S-R binding and related inhibition processes in alteration trials.

## Discussion

The present study was designed to examine top-down and bottom-up mechanisms underlying the influence of trial history on Stroop task performance and related brain activity. Stroop stimuli contain both relevant (i.e. colour) and irrelevant stimulus features (i.e. word meaning). Although participants were explicitly instructed to focus on the relevant colour of a word, they were not able to ignore the irrelevant word meaning. As a result, incongruent stimuli led to slower responses and higher error rates than congruent stimuli. The higher prevalence of incorrect responses related to word meaning supported that participants were not able to ignore irrelevant information. These behavioural differences were complemented by differences in ERPs. In accordance with previous studies, a negative going shift in the ERP was observed between 350–500 ms post-stimulus in incongruent trials compared to congruent trials, the so-called N450 component [19], [20]. Neural generators of the N450 have been localised in the anterior cingulate cortex (ACC), a brain structure implicated in attention and conflict control [10], [19], [34]. The more pronounced N450 in those trials in which conflict is expected to be most pronounced, supports that enhanced conflict indeed underlies the observed congruency effects [19], [35], [36]. However, it should be noted that the ERP differences between congruent and incongruent stimuli were rather small.

Trial level analysis also showed that performance efficiency declined with time on task and related brain activity showed a general reduction in P2 and P3 amplitude, which was most pronounced from the first to the second half hour of task performance. Both ERP components have been related to attention to task-relevant information [37], and the observed attenuation of these components confirmed the presumed negative influence of mental fatigue on attention mechanisms [1], [3], [12]. We hypothesised that due to fatigue-related deteriorations in attention, irrelevant information might interfere more strongly with information processing. However, the observed effects of mental fatigue were similar for congruent and incongruent stimuli. Previous findings showed that mental fatigue does affect selective attention mechanisms, however, the stimulus features defining relevance in these studies were related to spatial characteristics of the stimuli (selective attention task [1] and Simon task [12]), requiring spatial attention to select relevant information among distracters. The stimulus words in a Stroop task contain both relevant and irrelevant information within the same spatial location and therefore spatial attention is less critical during Stroop task performance. The results of the present study seem to indicate that spatial and non-spatial forms of attention are differentially affected by mental fatigue. The absence of differences in the effects of mental fatigue on congruent and incongruent Stroop words might also indicate that Stroop interference is mainly related to response conflict instead of conflict at a perceptual level. Previous studies indicated that although conflict detection was affected by mental fatigue [11], solving response-related conflicts was relatively unaffected by mental fatigue [38].

Of particular relevance in the present study was the influence of stimulus history on the processing of Stroop stimuli. It seems evident that people use available information to bias the forthcoming processing of information [39]. In Stroop task performance advance information provided by task instructions presented at the start of the experiment and information provided by stimuli presented in previous trials might be used to modulate specific functional processes executed within one of the information processing routes proposed in the dual route models. More specific, task instructions are expected to determine the stimulus-response translation rules used in the controlled route, while S-R binding, for example, is expected to have an influence on processing through the automatic route. The question addressed in this study is in fact twofold; first, which aspects of available information are used to bias functional processes, and second, how do these features modulate information processing within the top-down and bottom-up routes?

To start with the first part of the question, Botvinick et al. [9] explicitly argued that the occurrence of conflict is a crucial condition for adjustments in subsequent processing of information. This claim was further elaborated by Kerns et al. [8], who showed that greater ACC conflict-related activity on the previous trial was indeed associated with more pronounced adjustments in performance on the subsequent trial. Moreover, they observed enhanced PFC activity in subsequent trials with the greatest post-conflict behavioural adjustments, which is in line with the proposed role of the PFC in the actual implementation of top-down control of attention, namely biasing information processing towards relevant information [40]. Initial analysis showed that the behavioural data of this study were in agreement with the conflict adaptation perspective. Performance efficiency varied as a function of the level of conflict elicited in the previous trial, that is, larger interference effects were found on trials following a congruent Stroop stimulus than on trials following an incongruent stimulus. A similar pattern of results was observed regarding N450 amplitude, supporting enhanced conflict processing in the ACC in trials following congruent trials. Moreover, this analysis seemed to confirm that conflict elicited in previous trials indeed induced a stronger attention focus on task-relevant stimulus features and reduced interference of irrelevant stimulus information in subsequent trials (i.e. II trials were faster than CI trials), but also reduced facilitation produced by congruent irrelevant features (i.e. congruent word meaning) in congruent stimuli presented after conflict trials (i.e. IC trials were slower than CC trials). It is important to note that these effects were not related to a shift in speed–accuracy trade-off. Faster performance was not accompanied by increased error rates. Therefore, the results, so far, seem to indicate that conflict is indeed used to bias processing of upcoming information. Nonetheless, not all evidence is in line with this conclusion. Performance adjustments proposed by Botvinick et al. [9] consist of modulation of the attentional bias; a more adequate perceptual selection triggered by cognitive conflict would reduce interference of irrelevant information in the following trial. We found higher error rates in incongruent trials than in congruent trials and, as expected, an above average proportion of the incorrect responses in incongruent trials was related to the irrelevant word meaning. However, this pattern of results was independent of the level of conflict elicited in previous the trial, which is inconsistent with expectations based on the conflict monitoring model.

Based on findings that the conflict SP was particularly sensitive to characteristics of the previous trial [21], we expected effects of trial history in the 500–700 ms post-stimulus interval. However, in the present study no differences between congruent and incongruent trials were observed in this interval. The difference between results observed in the present study and the Larson et al. study might, at least partly, be related to differences in the level of conflict elicited by incongruent Stroop words; in the Larson et al. [21] study the proportion congruent stimuli was larger than the proportion incongruent stimuli (.7:.3), which was argued to increase the level of conflict elicited by incongruent stimuli. In the present study congruent and incongruent stimuli were presented with equal probability. The lower levels of conflict elicited in our paradigm might have reduced the demands placed on conflict related processing mechanisms and therefore resulted in lower SP amplitude.

The occurrence of conflict turned out not to be the only property of a stimulus involved in modulating subsequent performance. If conflict would be the most important requirement for tuning subsequent behaviour, smaller or even no changes in top down control are expected after congruent stimuli, since in these trials there is a match between information processed through the controlled and the automatic route and no conflict is elicited initiating top down control. The results showed the contrary, remarkable differences in both speed and accuracy were observed between congruent stimuli which were repetitions of previous congruent stimuli and congruent stimuli that had no features in common with the previous congruent stimulus, showing that the processing of Stroop stimuli was actually affected by preceding no-conflict stimuli. Not only in the CC trials we observed the advantage of repetition of the relevant colour feature on performance; responses in both congruent and incongruent colour-repeat trials were faster and more accurate than in other repetition conditions.

The feature integration model emphasizes that it is not solely the repetition of specific stimulus features that modulates consecutive performance. According to this model sequence effects are related to the reactivation of S-R bindings formed on a previous trial. Colour was directly associated with a specific response in our Stroop task and consistent with the predictions based on the feature integration model a clear advantage was observed on speed and accuracy in S-R repetition trials (i.e. colour-repeat trials) compared to the word-repeat conditions in which the response was not repeated. The results showed that if both colour and word were repeated, RTs were faster than in the colour-repeat condition, indicating that not only relevant features might have influenced repetition effects. However, the additional effect of repeating word features were relatively modest compared to sequence effects related to repetitions of relevant stimulus features, supporting the conclusion of Dutzi and Hommel [29] that the strength of S-R binding is dependent on stimulus relevance. Task relevant features and features that draw attention otherwise are bound more strongly than irrelevant features not attracting attention. In addition, error data supported that S-R binding was realised on previous trials and that these S-R associations had an effect on subsequent task performance. In line with our expectations, the results showed that the proposed inhibition of previous responses in stimulus alternation trials [29] resulted in a bias against the previous response resulting in a highly significant prevalence of responses which were not inhibited.

In addition to the observed behavioural results, the ERP data confirmed that repetition of relevant features facilitated processing of Stroop words; the parietal SP amplitude was smaller in colour-repeat trials than in the other repetition conditions. If indeed, as has been suggested, SP amplitude is related to conflict processing, the results suggest reduced conflict due to feature repetition. Alternatively, Chen and Melara [41] reported modulations of the SP in the absence of conflict adaptation, as is the case in our CC trials. They argued that SP effects reflected memory processes related to updating S-R associations in working memory instead of conflict adaptation. In our colour-repeat trials existing S-R association can be maintained, while in the other repetition conditions old S-R bindings have to be unbound and new associations have to be formed. Actually it seems more likely that this process is reflected in the enhanced SP amplitude observed in our results. At this moment it seems warranted to conclude that it is indeed S-R binding that creates a substantial performance benefit in Stroop task performance [22]–[24] - this repetition effect was even more than five times larger than conflict related sequence effects observed on response speed.

Trial level analysis showed a clear effect of mental fatigue on task performance and related brain activity. Essential is that these effects do not seem to differentiate across sequence conditions in general. Additional analyses taking repetition of stimulus features into account, however, did show differential effects of mental fatigue; the fatigue-related performance decline was found to be more pronounced in trials in which the irrelevant word feature or no features were repeated compared to colour-repeat trials. Given that automatic information processing is relatively unaffected by mental fatigue [2], this pattern of results confirms that repetition of relevant stimulus features and the formation of S-R associations mainly is performed via the automatic, bottom-up processing route. Thus the results of the present study show that S-R binding seems to have a more substantial influence on subsequent behaviour than the occurrence of conflict. Moreover, the three colour version of the Stroop task allowed the evaluation of conflict related adjustments on performance under three different repetition conditions (i.e. colour-, word- and no-repeat conditions. The results showed that conflict actually did not influence forthcoming behaviour if the relevant stimulus feature (and therefore also the response) was repeated and in case no features were repeated, that is, in complete alternation trials. The only condition in which conflict-related behavioural adjustments were observed was in the word-repeat condition; RTs in incongruent word-repeat trials were faster if the previous trial was congruent than if it was incongruent. Note that this pattern is opposite to that expected on base of the conflict monitoring model, according to which II trials are expected to be faster than CI trials. The conflict-related sequence effects on performance in the word-repeat condition were not supported by changes in related brain activity.

This does not mean, though, that trial-by-trial conflict monitoring does not play a role at all in modulating cortical processing of incoming information. The ERP data does show differences between CI_C+W−_ and II_C+W–_trials: incongruent colour repeat trials preceded by an incongruent trial elicited a larger P2 than trials following a congruent stimulus. This suggests a larger engagement of attention in processing of II_c+W−_ trials, which is in line with the conflict monitoring hypothesis [9], [37]. It should be noted, though, that these ERP-effects were not accompanied by changes in behaviour. Apparently, the benefit of additional top-down control elicited by a previous incongruent trial is relatively small compared to the effects of repeating a congruent S-R pair. Moreover, when considering the congruent trials, little effect of colour-word congruency of the previous trial is observed. However, stimulus-response incongruency (i.e. absence of colour repeat) did result in impaired performance, but was interestingly also associated with ERP effects typically associated with conflict detection (an increase in N2 magnitude [13], [16], [18]) and information processing impairment (a prolonged P3, indicating longer information processing). These effects were not modulated by time on task: stimulus-response conflict detection did not diminish over time.

Together, these results show that Stroop task performance depends on two sources: first, we do find some evidence confirming the conflict monitoring model proposed by Botvinick *et al*. [9], that is, observed conflict in a trial leads to increased attentional engagement on the next trial. However, we also show that the effects of top-down control are relatively small, and that Stroop task performance largely depends on stimulus-response binding: when a particular stimulus-response pair is repeated, this leads to a performance benefit. Non-repeats are associated with performance impairments, and ERP correlates of conflict detection, irrespective of colour-word (in)congruency. Moreover, stimulus-response effects are relatively independent of mental fatigue, suggesting trial history effects are independent of top-down control [2], [38].
